# Spatial organization meets temporal control: RNA-mediated localization of the cyanobacterial circadian clock

**DOI:** 10.1128/spectrum.01192-26

**Published:** 2026-07-21

**Authors:** Rakefet Schwarz

**Affiliations:** 1 The Mina and Everard Goodman Faculty of Life Sciences, Bar-Ilan University 108397 , Ramat-Gan, Israel; Reichman University, Herzeliya, Israel

**Keywords:** circadian clock, spatial organization, KaiB, Rbp2, RNA scaffolding, cyanobacteria, *Synechococcus elongatus*

## Abstract

The cyanobacterial circadian clock, the only rigorously established prokaryotic circadian system, is driven by the KaiABC core oscillator and accompanied by dynamic changes in KaiA and KaiC localization. Because KaiB had not previously been imaged in living cells, the spatial organization of the complete core oscillator remained unresolved. Recent work by H. J. Bevir, C. C. Hooper, P. Saikley, T. Chaljian, et al. (Microbiol Spectr 14:e01845-25, 2026, https://doi.org/10.1128/spectrum.01845-25) shows that KaiB localizes to the cell poles at night, completing the spatial model of the cyanobacterial clock. Using a frankenbody-based imaging strategy in bacteria for the first time, the authors overcome longstanding barriers to visualizing KaiB *in vivo*. They also demonstrate that the RNA-binding protein Rbp2 co-localizes with KaiB and KaiC, and that its RNA-binding activity is required for robust localization. These results support a model in which RNA-mediated scaffolding assembles a nighttime polar clock complex, providing substantial advancement in understanding clock mechanism and emphasizing the importance of spatial organization in circadian robustness.

## COMMENTARY

Circadian clocks are traditionally conceptualized as biochemical oscillators, with timing encoded in cycles of protein modification and interaction. In cyanobacteria, the only prokaryotes with a thoroughly established circadian clock, the KaiABC system represents an example of a post-translational circadian oscillator that can function *in vitro* (1). It has also become evident that temporal regulation is inseparable from spatial organization (2).

The study by Bevir et al. demonstrates, for the first time, the polar localization at night of the oscillator core component KaiB; moreover, it is shown that the RNA-binding protein Rbp2 is required for robust spatial organization (3).

Previous work established that KaiA and KaiC transition from a diffuse cytoplasmic distribution during the day to polar foci at night, suggesting that subcellular localization may contribute to clock function (4). However, technical challenges stemming from impairment of KaiB function when fused to a fluorescent protein prohibited the determination of KaiB localization. Bevir et al. overcome this limitation by elegantly implementing “frankenbodies,” genetically encoded intracellular single-chain variable fragments that bind epitope-tagged proteins. Using this approach for the first time in prokaryotes, KaiB was visualized without disrupting its function, a critical advance given KaiB’s sensitivity to structural perturbation (3). The authors demonstrate that KaiB mirrors the localization dynamics of KaiA and KaiC: diffuse during the day and concentrated at the cell poles at night (3). This finding completes the spatial picture of the core oscillator and strongly supports the existence of a nighttime polar clock complex. The co-localization of KaiB with KaiC further reinforces the biochemical model in which KaiB associates with phosphorylated KaiC during the dephosphorylation phase of the cycle (5).

Additionally, Rbp2, previously identified as part of an extended clock network, is shown here to exhibit similar circadian localization dynamics and to co-localize with KaiC at the poles at night (3, 6). Importantly, the authors demonstrate that mutations disrupting Rbp2’s RNA-binding domain markedly reduce its ability to form polar foci (3).

The authors propose a model in which Rbp2, in complex with the KaiABC oscillator, binds to an RNA that localizes, along with the protein complex, to the pole of the cell (3). The spatial organization described by Bevir et al. may serve to concentrate clock proteins at the pole and enhance interaction, particularly when protein abundance is low, as occurs late at night (7). Moreover, spatial segregation may help coordinate downstream processes, such as chromosome compaction or cell division, which are known to be under circadian control (8).

An additional intriguing observation is the presence of multiple foci in a subset of cells (3). This raises the possibility that clock complexes may assemble at multiple sites before coalescing at the pole, or that multiple functional units coexist. Such heterogeneity could reflect stochastic aspects of protein assembly or regulated subpopulations of complexes with distinct functions.

The methodological advances in this study are also noteworthy. The successful application of frankenbodies in bacteria opens new possibilities for imaging proteins that are incompatible with traditional fluorescent fusions (3). This approach may prove broadly useful for studying dynamic protein localization in prokaryotes, particularly for small or conformationally sensitive proteins. Furthermore, frankenbodies may conceptually be used to interfere with protein function *in vivo*.

Despite these advances, several key questions remain. What specific RNA species are involved in Rbp2-mediated localization? Are these transcripts themselves under circadian regulation, and do they exhibit spatial dynamics? Work on cyanobacterial mRNA localization, such as the spatial control of photosynthetic transcripts, provides a useful precedent (9). Furthermore, how is the transition between diffuse and localized states regulated at the molecular level?

Another important avenue for future work is to determine whether spatial organization feeds back on the biochemical oscillator. While the KaiABC system can oscillate *in vitro* without spatial cues (1), the *in vivo* environment is far more complex. It is plausible that localization enhances robustness, synchrony, or responsiveness to environmental signals.

In summary, Bevir et al. provide a significant advance in our understanding of the cyanobacterial circadian clock by linking protein localization, RNA binding, and oscillator function (3). Their findings support a model in which the clock is a spatially organized biochemical oscillator complex, with RNA playing a central scaffolding role.

## References

[1] Rust MJ, Golden SS, O’Shea EK. 2011. Light-driven changes in energy metabolism directly entrain the cyanobacterial circadian oscillator. Science 331:220–223. doi:10.1126/science.1197243 21233390 PMC3309039

[2] Fang M, Partch CL, LiWang A, Golden SS. 2025. Prokaryotic circadian systems: cyanobacteria and beyond. Annu Rev Microbiol 79:523–545. doi:10.1146/annurev-micro-041222-022934 40858299

[3] Bevir HJ, Hooper CC, Saikley P, Chaljian T, Lin Y, Cohen SE. 2026. Circadian clock proteins KaiB and Rbp2 of *Synechococcus elongatus* display oscillations in their subcellular localization patterns. Microbiol Spectr 14:e0184525. doi:10.1128/spectrum.01845-25 41451986 PMC12889050

[4] Cohen SE, Erb ML, Selimkhanov J, Dong G, Hasty J, Pogliano J, Golden SS. 2014. Dynamic localization of the cyanobacterial circadian clock proteins. Curr Biol 24:1836–1844. doi:10.1016/j.cub.2014.07.036 25127213 PMC4139467

[5] Chang Y-G, Cohen SE, Phong C, Myers WK, Kim Y-I, Tseng R, Lin J, Zhang L, Boyd JS, Lee Y, Kang S, Lee D, Li S, Britt RD, Rust MJ, Golden SS, LiWang A. 2015. A protein fold switch joins the circadian oscillator to clock output in cyanobacteria. Science 349:324–328. doi:10.1126/science.1260031 26113641 PMC4506712

[6] McKnight BM, Kang S, Le TH, Fang M, Carbonel G, Rodriguez E, Govindarajan S, Albocher-Kedem N, Tran AL, Duncan NR, Amster-Choder O, Golden SS, Cohen SE. 2023. Roles for the *Synechococcus elongatus* RNA-binding protein Rbp2 in regulating the circadian clock. J Biol Rhythms 38:447–460. doi:10.1177/07487304231188761 37515350 PMC10528358

[7] Chew J, Leypunskiy E, Lin J, Murugan A, Rust MJ. 2018. High protein copy number is required to suppress stochasticity in the cyanobacterial circadian clock. Nat Commun 9:3004. doi:10.1038/s41467-018-05109-4 30068980 PMC6070526

[8] Markson JS, Piechura JR, Puszynska AM, O’Shea EK. 2013. Circadian control of global gene expression by the cyanobacterial master regulator RpaA. Cell 155:1396–1408. doi:10.1016/j.cell.2013.11.005 24315105 PMC3935230

[9] Mahbub M, Hemm L, Yang Y, Kaur R, Carmen H, Engl C, Huokko T, Riediger M, Watanabe S, Liu L-N, Wilde A, Hess WR, Mullineaux CW. 2020. mRNA localization, reaction centre biogenesis and thylakoid membrane targeting in cyanobacteria. Nat Plants 6:1179–1191. doi:10.1038/s41477-020-00764-2 32895528

